# The complete mitochondrial genome of pitted stingray *Bathytoshia brevicaudata* (Myliobatiformes: Dasyatoidea)

**DOI:** 10.1080/23802359.2020.1829121

**Published:** 2020-10-09

**Authors:** Jong-Oh Kim, Yong Bae Seo, Jiyoung Shin, Ji-Young Yang, Gun-Do Kim

**Affiliations:** a Institute of Marine Biotechnology, Pukyong National University, Busan, Republic of Korea; b Department of Food Science & Technology, Pukyong National University, Busan, Republic of Korea; c Department of Microbiology, Pukyong National University, Busan, Republic of Korea

**Keywords:** *Bathytoshia brevicaudata*, Dasyatoidea, mitochondrion genome, phylogenetic analysis

## Abstract

The complete mitochondrial genome of pitted stingray, *Bathytoshia brevicaudata* (Myliobatiformes: Dasyatoidea) was investigated by next-generation sequencing. The analyzed mitochondrial genome was 17,640 nucleotides in length and had 59.2% for AT contents. This genome contains 2 ribosomal RNA genes, 13 protein-coding genes, 22 transfer RNA genes. and 1 putative control region. Five protein-coding genes (ATPase6, COII, ND2, ND3, ND4) including incomplete stop codons and four tRNAs have atypical codons. The phylogenetic inference including 13 species of the same family revealed a close relationship with *Pteroplatytrygon violacea*. This is the first mitochondrial genome report from genus Bathytoshia.

The phylogenetic relationships of the stringrays have been the issue since the first modern morphological classification by Compagno (1973). Stingrays are a group of sea rays and common in coastal tropical and subtropical marine environment (Carpenter and Niem 1999). In Korea, pitted stingray *Bathytoshia brevicaudata* is commonly captured from all coastal area. However, there is not enough biological information or genetic studies on pitted stingray. Here, we determined the complete mitochondrial genome sequence of *B. brevicaudata* compared with other mitogenome data reported previously.

An individual sample was collected at the East Sea in Korea (37°32′55.4″N 129°07′59.4″E). The voucher specimen (Sample no. MFDS-FHO10) was deposited at Department of Food Engineering, Pukyong National University. Total genomic DNA was extracted using the DNeasy Blood and Tissue Kit (Qiagen, Germany) and DNA library was constructed using MGIEasy DNA Library Prep Kit (MGI, China). DNA sequencing was carried out using MGISEQ-2000 platform and the sequence reads were mapped to reference sequence (Accession No. NC021132) using Geneious Prime 2020.1.2 (Biomatters, New Zealand) after trimming the adaptor sequence with Cutadapt 1.9 (default parameters) (Martin 2011). The annotation and phylogenetic tree of the mitogenome sequence were conducted by MitoFish (Sato et al. 2018) and MEGA X (Maximum Likelihood methods) (Kumar et al. 2018), respectively.

The assembled circular mitogenome was 17,640 nucleotides in length and had 59.2% for AT contents. This genome contains two ribosomal RNA genes, 13 protein-coding genes, 22 transfer RNA genes and one putative control region. Five protein coding genes (ATPase6, COII, ND2, ND3, ND4) were including incomplete stop codons and tRNAs for Leucine and Serine have atypical codons. All these information and assembled sequence were submitted to GenBank with accession number MT780270. In addition, the raw sequencing data were deposited in SRA (SRA no. PRJNA657136).

The phylogenetic analysis with previously reported 13 mitogenome sequences from Dasyatoidae showed that *B. brevicaudata* is closely related to the *Pteroplatytrygon violacea* (Accession No. NC024570) (Figure 1). This result is consistent with the previous report on the family Dasyatidae based on the NADH2 gene (Last et al. 2016). This is the first mitochondrial genome report from the genus Bathytoshia, therefore this study will contribute to understand the molecular phylogeny of the family Dasyatoidae.

**Figure 1.**
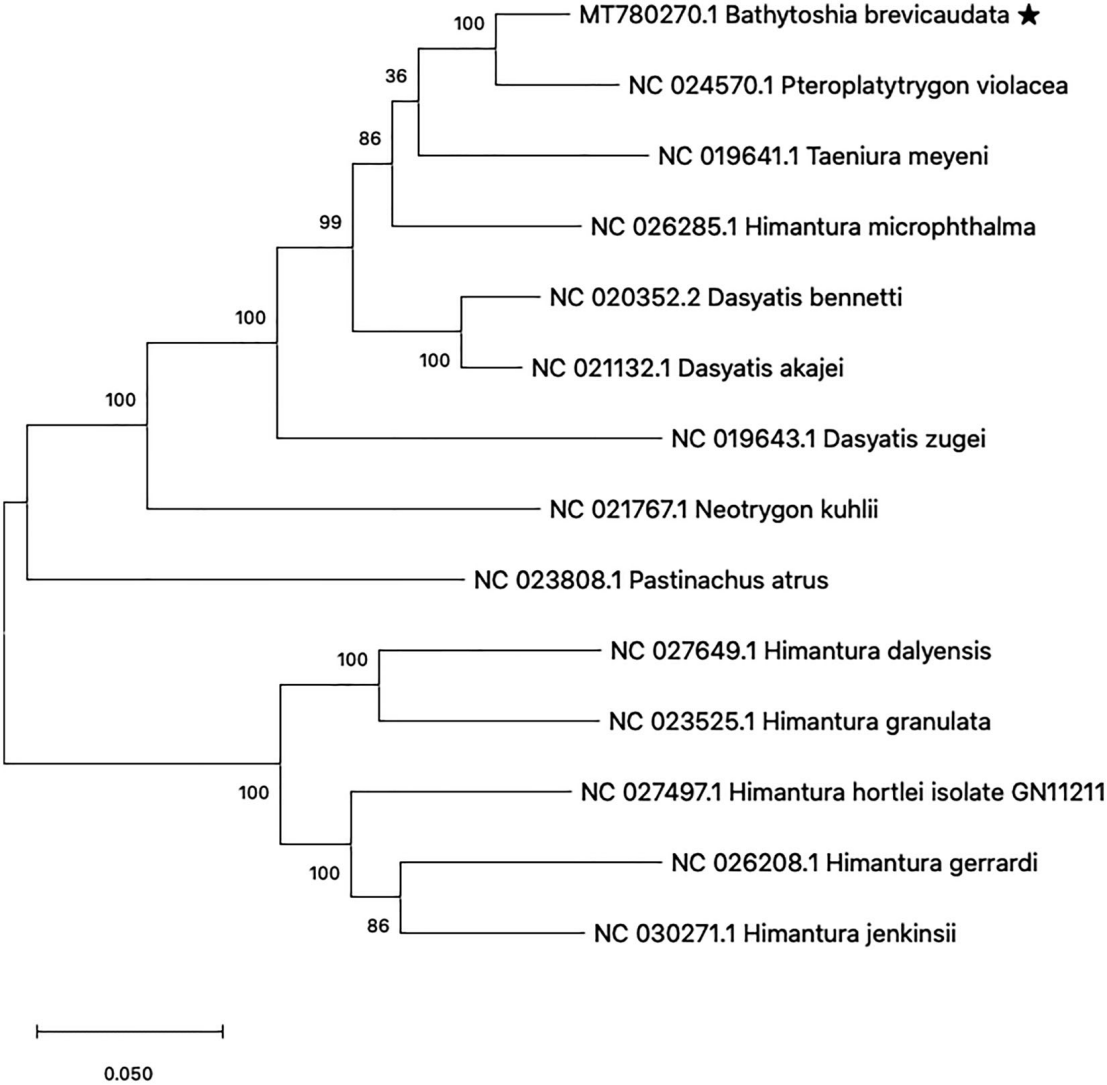
Phylogenetic analysis of 14 species in family Dasyatoidae. The previously reported 13 species and *Bathytoshia brevicaudata* mitogenome sequence were aligned using ClustalW and phylogenetic tree was constructed by maximum likelihood method with 1000 bootstrap. The percentage at each node is the bootstrap probability.

## Data Availability

Mitogenome data supporting this study are openly available in NCBI: GenBank Accession Numbers - https://www.ncbi.nlm.nih.gov/nuccore/MT780270 and Associated BioProject - https://www.ncbi.nlm.nih.gov/bioproject/PRJNA657136.
